# Effect of Concomitant Use of Analgesics on Prognosis in Patients Treated With Immune Checkpoint Inhibitors: A Systematic Review and Meta-Analysis

**DOI:** 10.3389/fimmu.2022.861723

**Published:** 2022-05-06

**Authors:** Ziyang Mao, Xiaohui Jia, Panpan Jiang, Qinyang Wang, Yajuan Zhang, Yanlin Li, Xiaolan Fu, Min Jiao, Lili Jiang, Zhiyan Liu, Hui Guo

**Affiliations:** ^1^ Department of Medical Oncology, The First Affiliated Hospital of Xi’an Jiaotong University, Xi’an, China; ^2^ Department of Respiratory and Critical Care Medicine, Respiratory and Critical Care Medicine, The Affiliated Hospital of Northwest University, Xi’an No. 3 Hospital, Xi’an, China; ^3^ Centre for Translational Medicine, The First Affiliated Hospital of Xi’an Jiaotong University, Xi’an, China; ^4^ Key Laboratory of Environment and Genes Related to Diseases, Xi’an Jiaotong University, Ministry of Education of China, Xi’an, China

**Keywords:** immune checkpoint inhibitors, analgesics, drug–drug interactions, prognosis, meta-analysis

## Abstract

**Background:**

Drug–drug interactions (DDIs) pose new challenges beyond traditional pharmacodynamics in the context of optimizing the treatment options with immune checkpoint inhibitors (ICIs). To alleviate cancer-related pain, analgesics are of absolute vital importance as chronic medications used by cancer patients. However, the possible outcome of ICI treatment concomitant with analgesics remains unclear.

**Methods:**

Original articles describing the possible influence of analgesics use on ICI treatment published before December 1, 2021 were retrieved from PubMed, Embase, and the Cochrane Library. Odds ratio (OR) with 95% confidence interval (CI) for objective response rate (ORR), hazard ratio (HR) with 95% CI for progression-free survival (PFS), and overall survival (OS) were calculated using the random-effects or fixed-effects model, and heterogeneity was assessed using the *χ* ^2^-based *Q*-test. Publication bias was examined by funnel plot analysis.

**Results:**

A total of 11 studies involving 4,404 patients were included. The pooled OR showed that opioid use decreased the response of opioid users to ICIs compared to non-opioid users (OR = 0.49, 95% CI = 0.37–0.65, *p* < 0.001). Compared to patients who did not receive opioids, opioid users had an increased risk of progression and mortality (HR = 1.61, 95% CI = 1.37–1.89, *p* < 0.001; HR = 1.67, 95% CI =1.30–2.14, *p* < 0.001, respectively). Furthermore, the concomitant use of non-steroidal anti-inflammatory drugs (NSAIDs) was not significantly associated with differences in ORR, PFS, and OS in patients treated with ICIs (OR = 1.40, 95% CI = 0.84–2.32, *p* = 0.190; HR = 0.90, 95% CI = 0.77–1.06, *p* = 0.186; HR = 0.90, 95% CI = 0.71–1.14, *p* = 0.384, respectively).

**Conclusion:**

The concomitant use of opioids during ICI treatment has an adverse effect on patient prognosis, while the use of NSAIDs is not significantly associated with the prognosis in patients treated with ICIs.

## Introduction

Drug–drug interactions (DDIs) are an important concern in the context of anticancer therapy due to the narrow therapeutic index and inherent toxicity of anticancer agents (1). Concomitant medications can affect the efficacy of systemic therapy through their effects on pharmacodynamics, including changes in drug absorption, distribution, metabolism, or elimination (1). Recently, DDIs have posed new challenges beyond traditional pharmacodynamics in the era of the rapid development of immune checkpoint inhibitors (ICIs). By blocking cytotoxic T-lymphocyte-associated 4 (CTLA-4) and programmed cell death protein/ligand 1 (PD-1/PD-L1), ICIs can activate the anticancer function of exhausted T cells and can elicit remarkable survival benefits to patients with multiple malignancies (2). However, ICIs are not consistently effective across different individuals (2). Although tumoral PD-L1 expression has been evaluated to predict the response to ICI therapy to a certain extent in clinical practice, it is not an entirely reliable biomarker (3). For instance, among the melanoma patients who failed to respond to ICI therapy, 48%–56% of patients were positive for PD-L1 expression (4, 5). Another conspicuous biomarker, the tumor mutation burden (TMB) is considered to be positively correlated with the efficacy of ICI therapy (6). Nevertheless, the results of the KEYNOTE-158 trial showed that there was no significant difference in prognosis between the TMB-high and TMB-low cohort among patients with advanced solid tumors receiving pembrolizumab (7). Considering these cases, exploring additional factors influencing efficacy of ICIs is urgent, among which DDI has received considerable attention from researchers.

Several studies have highlighted the link between DDI and the response to ICI therapy. Steroids, antibiotics, and proton pump inhibitors (PPIs) have been demonstrated to influence prognosis in patients treated with ICIs compared to those receiving ICI treatment (8–10). Furthermore, Cortellini et al. demonstrated worse outcomes in patients receiving steroids or PPIs might be attributed to adverse disease features, while the impact of antibiotics on clinical outcomes is presumably a consequence of immune modulation (11). Conversely, Ni et al. revealed that statins can promote anticancer immunity by downregulating PD-L1 expression (12). Accordingly, a retrospective cohort study confirmed that administration of statins during ICI treatment was associated with improved prognosis in patients with malignant pleural mesothelioma or advanced non-small cell lung cancer (NSCLC) (13). Thus, DDIs play an important role in the influence of concomitant medications on the efficacy of ICIs.

In addition to the above medications, analgesics use is also critical to cancer patients. Approximately 30%–50% of cancer patients will experience moderate to severe pain, usually at multiple sites, with different etiologies and potential mechanisms (14). As for the therapeutic approach, the World Health Organization (WHO) recommends an analgesic ladder based on pain intensity [i.e., non-steroidal anti-inflammatory drugs (NSAIDs) for mild pain leading up to strong opioids for severe chronic pain] (15). However, the possible influence of DDIs between ICIs and analgesics on the efficacy of ICIs, including opioids and NSAIDs, remains unclear and lacks clinical evidence. Many preclinical studies have highlighted that opioids can promote tumor progression and metastasis directly as opioid receptors are overexpressed in several tumors (16, 17), which, in turn, may impair the response to treatment with ICIs. Furthermore, opioids can suppress the immune system in various ways, such as by affecting T-cell function, upregulating regulatory T cells (Tregs), and interfering with the composition of the intestinal microbiota that damages the entire immune system (18–21). Conversely, *via* their inhibition of cyclooxygenase-2 (COX-2), NSAIDs may be beneficial for the treatment of ICIs, since overexpression of COX-2 has been found in a wide range of tumors, and is associated with malignant tumor phenotypes and negative regulation of anticancer immunity (22–25).

Several retrospective cohort studies have explored the association of analgesics use with the efficacy of ICIs in advanced cancer patients (26–36). Most evidence suggests that opioids could cause a poor prognosis in patients treated with ICIs, while NSAIDs do not weaken the efficacy of ICIs. However, these are all retrospective cohort studies with a small sample size and conclusions are not sufficiently convincing. The exact effect of analgesics on ICI treatment deserves to be further explored, with due consideration on both pain alleviation and efficacy of ICI treatment, which are crucial for optimizing benefits to patients. Therefore, additional higher-level evidence-based research is needed to dispel doubts and better guide clinical practice. Given the above evidence, we conducted this meta-analysis to determine the effect of concomitant use of analgesics on outcomes in patients receiving treatment with ICIs.

## Materials and Methods

This meta-analysis was performed in accordance with the preferred reporting items for systematic reviews and meta-analyses (PRISMA) guidelines (37). We designed a formal protocol for this meta-analysis, which was registered in the Prospective Register of Systematic Reviews (PROSPERO CRD42021288940).

### Search Strategy

We systematically conducted an electronic search using PubMed, Embase, and the Cochrane Library to identify potentially relevant studies. Results from International conferences, such as the American Society of Clinical Oncology (ASCO), the European Society of Medical Oncology (ESMO), and the American Association for Cancer Research (AACR) were also selected to avoid any loss of information. Studies were identified using free text including the following terms: neoplasm, malignancy, ICIs, anti-PD1, anti-PD-L1, opioids, and NSAIDs, as well as specific drug names. The search was limited to studies published in English and published before December 1, 2021. Two authors (ZM and XJ) established the comprehensive search strategy, which is presented in **Table S1**.

### Inclusion and Exclusion Criteria

A study was included when all the following criteria were met: (a) involved patients with solid tumors or hematological malignancy treated with ICIs; (b) explored the DDI between ICIs and analgesics (opioids and NSAIDs); (c) involved primary endpoints, such as objective response rate (ORR), progression-free survival (PFS), and overall survival (OS); and (d) provided sufficient data to calculate the odds ratio (OR) or hazard ratio (HR) with 95% confidence interval (CI). Accordingly, the exclusion criteria were as follows: (a) lack of related or sufficient data; (b) designed as single-arm or dosage-finding studies; and (c) published as a meta-analysis, editorial, review, or case report. Two authors (ZM and XJ) checked the studies, and disagreements were decided by two senior investigators (HG and ZL).

### Data Extraction and Quality Assessment

The studies that met the inclusion criteria were selected and analyzed by two authors (PJ and QW). The following elements were extracted for each included study: first author’s surname, publication year, country of origin, sample size, type of concomitant analgesics received, type of study design, analysis, cancer type, ICIs type, line of treatment, median PFS and OS, OR for ORR with 95% CI, HR for PFS, and OS with 95% CI. Discrepancies were solved by the other two authors (YZ and YL).

Three independent authors (XF, MJ, and LJ) assessed the quality of the included studies by using the Quality Assessment of Newcastle–Ottawa Scale (NOS) for cohort studies (38). Discrepancies were resolved through discussion among all researchers. The total scores ranged from 0 (worst) to 9 (best), and NOS scores >6 indicated high-quality studies (38).

### Statistical Analysis

ORs with 95% CI for ORR from included studies were utilized to calculate the pooled OR. HRs with 95% CI for PFS or OS were synthesized in this meta-analysis. ORR was defined as proportion of complete response (CR) and partial response (PR) according to the Response Evaluation Criteria in Solid Tumors (RECIST) criteria (version 1.1) (39). PFS was defined as the time from initiation of ICIs to the date of disease progression or death from any cause, while OS was defined as the time from initiation of ICI treatment to the date of death from any cause. The heterogeneity of the pooled results was evaluated using the *χ* ^2^-based *Q*-test and quantified using the *I* ^2^ test. If *p* was < 0.10 for the *Q*-test or *I* ^2^ was >50%, we recognized significant heterogeneity, and the random-effects model was utilized to synthesize the data. Otherwise, the fixed-effects model was adopted (40). Subgroup analysis was performed mainly according to the cancer type and ICI type. Funnel plots were constructed to evaluate publication bias. Sensitivity analysis was used to examine the stability of the outcome. All statistical tests were two-sided and a *p-*value < 0.05 was considered statistically significant. Review Manager, version 5.3 (The Nordic Cochrane Centre, The Cochrane Collaboration, Copenhagen, Denmark) was used for all pooled analysis and GraphPad Prism, version 9.0.0 (GraphPad Software Inc., La Jolla, CA, USA) was used to construct graphical charts.

## Results

### Selection of Eligible Studies

**Figure 1:**
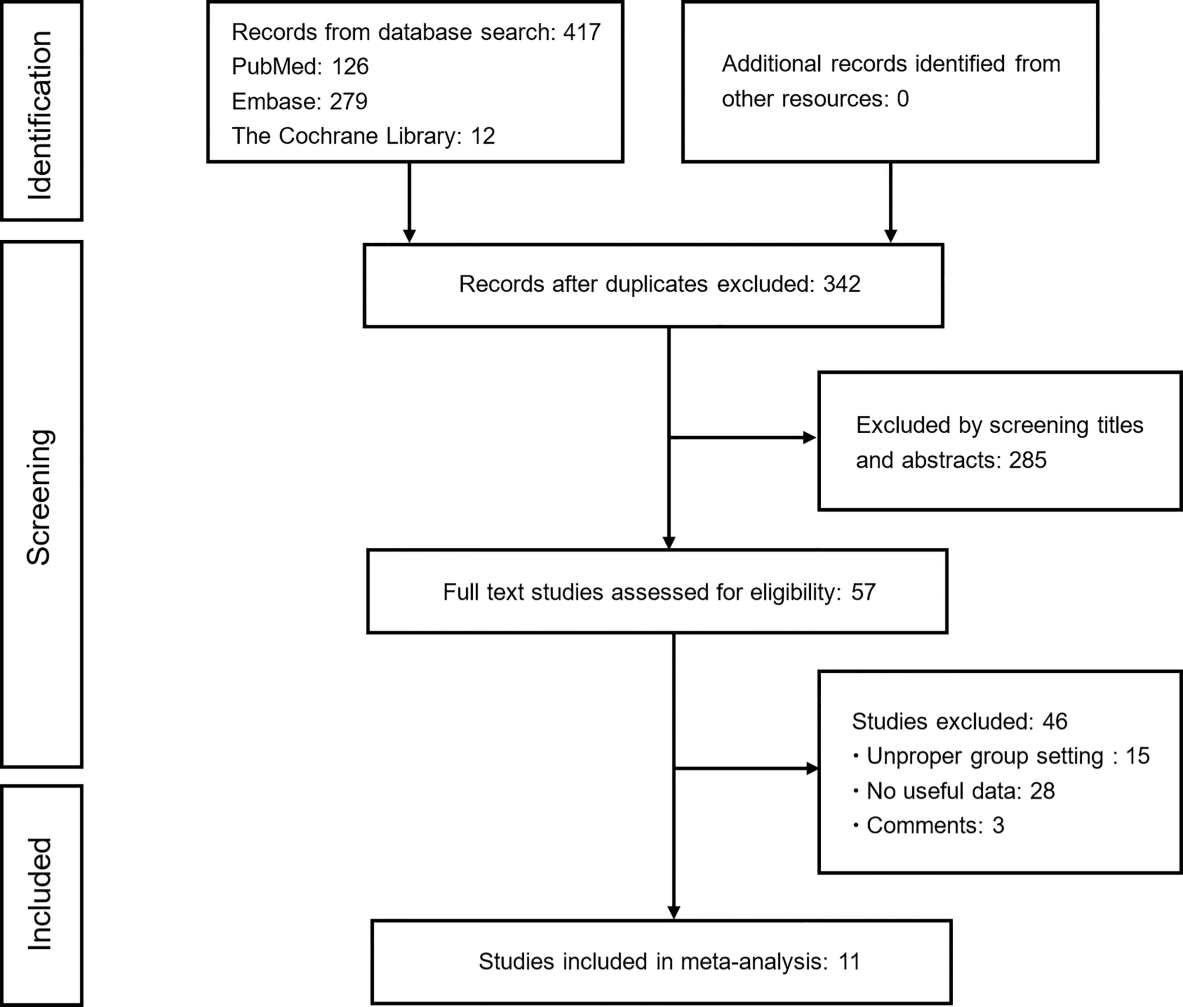
Flowchart of study selection. Based on the inclusion and exclusion criteria, 11 studies were included in this study.

**Figure 1** shows the flowchart used for the identification of eligible studies and the inclusion and exclusion criteria. A total of 417 studies were identified from the electronic databases. After removing duplicates and screening titles and abstracts, 57 potentially relevant studies remained for further eligibility evaluation. After a complete evaluation, 46 ineligible studies were excluded and 11 studies that explored the effect of concomitant analgesics on the survival of patients receiving ICIs were ultimately included in this meta-analysis.

### Characteristics of the Included Studies

All included studies (26–36) were retrospective, and a total of 4,404 participants were enrolled. Among them, two studies (31, 35) evaluated both NSAIDs and opioids, five studies (30, 32–34, 36) that only evaluated NSAIDs, and four studies (26–29) only evaluated opioids. Four studies (27, 30, 31, 33) only included patients with NSCLC and two studies (34, 36) only included melanoma patients. The remaining studies (26, 28, 29, 32, 35) included both NSCLC and melanoma or other types of cancer. Additional characteristics of these studies are listed in **Table 1**. The quality of these studies quantified by the NOS criteria ranged from 6 to 9, showing that all studies were of high quality and qualified for analysis. Details of the quality assessment are shown in **Table S2**.

**Table 1:** Baseline characteristics of studies included in the meta-analysis.

Concomitant medications	Study (years)	Country	Study design	Cancer type	ICIs treatment	Line of ICIs treatment	Patients (n) (Users/non-users)	Outcome	ORR(%) (Users vs non-users)	mPFS (months) (Users vs non-users)	mOS (months) (Users vs non-users)	Type of analysis
Opioids	Iglesias−Santamaría et al., 2020 (28)	Spain	Retrospective	Melanoma, NSCLC, Others	Atezo, Nivo, Pembro, Nivo + Ipi	≥1	55/47	PFS/OS	NA	4.5 vs 8.1	8.6 vs 26.3	Multivariate
	Cortellini et al., 2020 (35)	Italy	Retrospective	Melanoma, NSCLC, Others	Atezo, Nivo, Pembro, Others	≥1	68/944	ORR/PFS/OS	32.2 vs 38.6	NA	NA	Multivariate
	Taniguchi et al., 2020 (27)	Japan	Retrospective	NSCLC	Nivo	≥1	38/38	ORR/PFS/OS	2.6 vs 21.1	1.2 vs 2.1	4.2 vs 9.6	Univariate
	Botticelli et al., 2021 (26)	Italy	Retrospective	Melanoma, NSCLC, Others	Atezo, Nivo, Pembro, Avelu	≥1	42/151	ORR/PFS/OS	31.0 vs 52.3	3.0 vs 19.0	4.0 vs 35.0	Multivariate
	Miura et al., 2021 (31)	Japan	Retrospective	NSCLC	Nivo, Pembro	≥1	114/186	ORR/OS	13.9 vs 26.9	NA	5.7 vs 15.9	Multivariate
	Gaucher et al., 2021 (29)	France	Retrospective	Melanoma NSCLC Others	Ipi, Nivo, Pembro, Nivo + Ipi	1/2/3	173/199	ORR/PFS/OS	16.2 vs 33.7	NA	8.5 vs 29.4	Multivariate
NSAIDs	Failing et al., 2016 (36)	US	Retrospective	Melanoma	Ipi	1	31/128	ORR/PFS/OS	71.0 vs 64.1	NA	NA	Multivariate
	Cortellini et al., 2020 (35)	Italy	Retrospective	Melanoma, NSCLC, Others	Atezo, Nivo, Pembro, Others	≥1	59/953	ORR/PFS/OS	27.3 vs 38.2	NA	NA	Multivariate
	Wang et al., 2020 (34)	Multicountry	Retrospective	Melanoma,	Nivo, Pembro	1	122/208	ORR/PFS/OS	43.4 vs 41.3	8.5 vs 5.2	25.7 vs 27.3	Univariate
	Svaton et al., 2020 (33)	Czech	Retrospective	NSCLC	Nivo	1/2/3/4/5/6	45/178	PFS/OS	33.3 vs 28.1	6.9 vs 5.3	16.8 vs 12.8	Multivariate
	Wang et al., 2020 (32)	US	Retrospective	Melanoma, NSCLC	PD-1/L1 inhibitors, CTLA-4 inhibitors	NA	Melanoma: 32/58 NSCLC: 20/17	ORR/OS	Melanoma: 59.3 vs 19.0 NSCLC: 75.0 vs 35.3	NA	Melanoma: 25.4 vs 22.1 NSCLC: 37.7 vs 14.3	Multivariate
	Miura et al., 2021 (31)	Japan	Retrospective	NSCLC	Nivo, Pembro	≥1	140/160	ORR/OS	18.6 vs 27.5	NA	8.8 vs 15.9	Multivariate
	Kanai et al., 2021 (30)	Japan	Retrospective	NSCLC	Atezo, Nivo, Pembro	2/3	65/133	ORR/PFS/OS	20.0 vs 12.0	3.45 vs 3.94	7.85 vs 15.11	Univariate

ORR, objective response rate; mPFS, median progression-free survival; mOS, median overall survival; NSCLC, non-small cell lung cancer; NSAIDs, non-steroidal anti-inflammatory drugs; Ipi, Ipilimumab; Atezo, Atezolizumab; Nivo, Nivolumab; Pembro, Pembro- lizumab; Avelu, Avelumab; PD-1/L1, programmed cell death protein/ligand 1; CTLA-4, T-lymphocyte-associated 4; NA, not available.

### Concomitant Use of Opioids on ICIs Efficacy

**Figure 2:**
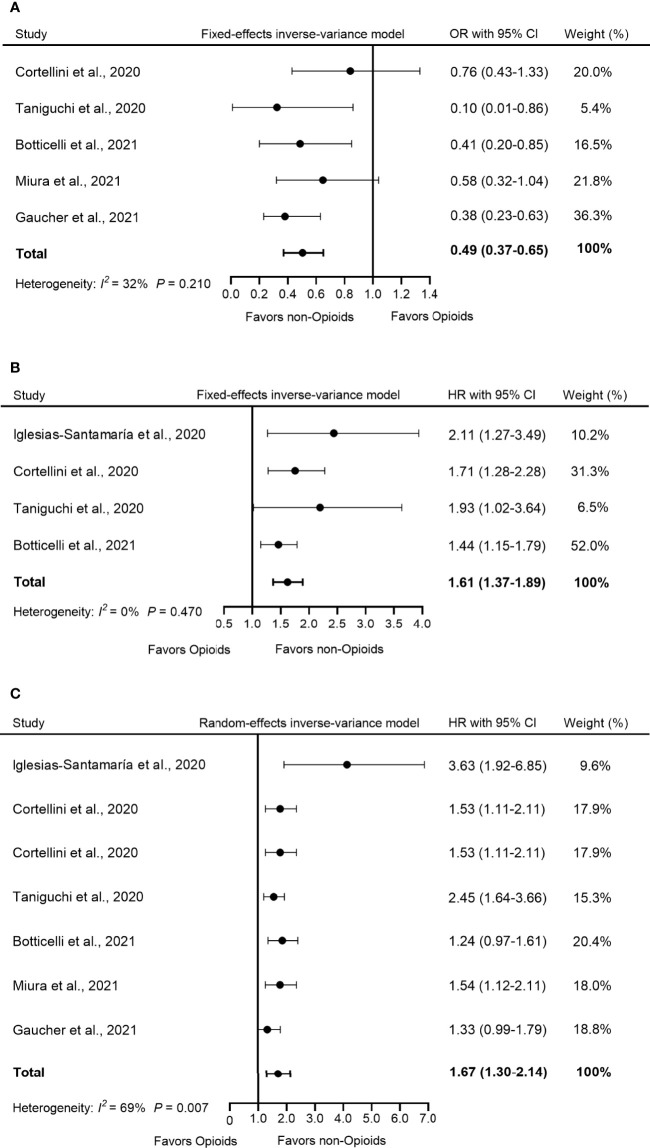
Forest plot of the correlation between concomitant use of opioids and **(A)** ORR, **(B)** PFS, and **(C)** OS in patients receiving ICIs. The pooled OR of ORR was 0.49 (95% CI = 0.37–0.65, *p* < 0.001) and the fixed-effects model was adopted. The pooled HR of PFS was 1.61 (95% CI = 1.37–1.89, *p* < 0.001) and the fixed-effects model was adopted. The combined HR of OS was 1.67 (95% CI = 1.30–2.14, *p* < 0.001) and the random-effects model was adopted. By definition, OR > 1 or HR < 1 implied a better prognosis for opioid users. ORR, objective response rate; PFS, progression-free survival; OS, overall survival; OR, odds ratio; HR, hazard ratio; CI, confidence interval; ICIs, immune checkpoints inhibitors.

Five studies reported the influence of opioid use on ORR in patients treated with ICIs. The pooled OR showed that the use of opioids decreased the response of opioid users to ICI treatment compared to non-opioid users (OR = 0.49, 95% CI = 0.37–0.65, *p* < 0.001) (**Figure 2A**). Furthermore, low heterogeneity was detected in the heterogeneity test (*I* ^2^ = 32%, *Q*-test *p* = 0.210).

Four studies used PFS as an indicator of outcome. The analysis of this study showed that compared to non-opioid users, the use of opioids increased the risk of progression by 61% among opioid users (HR = 1.61, 95% CI = 1.37–1.89, *p* < 0.001) (**Figure 2B**). Furthermore, no significant heterogeneity was observed in these studies (*I* ^2^ *=* 0%, *Q*-test *p* = 0.470).

OS data were available in six studies. Because the heterogeneity test showed a high level of heterogeneity (*I* ^2^ *=* 69%, *Q*-test *p* = 0.007) in these studies, we used a random-effects model for analysis. The pooled data for HR showed that the concomitant use of opioids was significantly associated with a poorer OS in patients receiving ICIs (HR = 1.67, 95% CI =1.30–2.14, *p* < 0.001) (**Figure 2C**).

### Concomitant Use of NSAIDs on the Efficacy of ICIs

**Figure 3:**
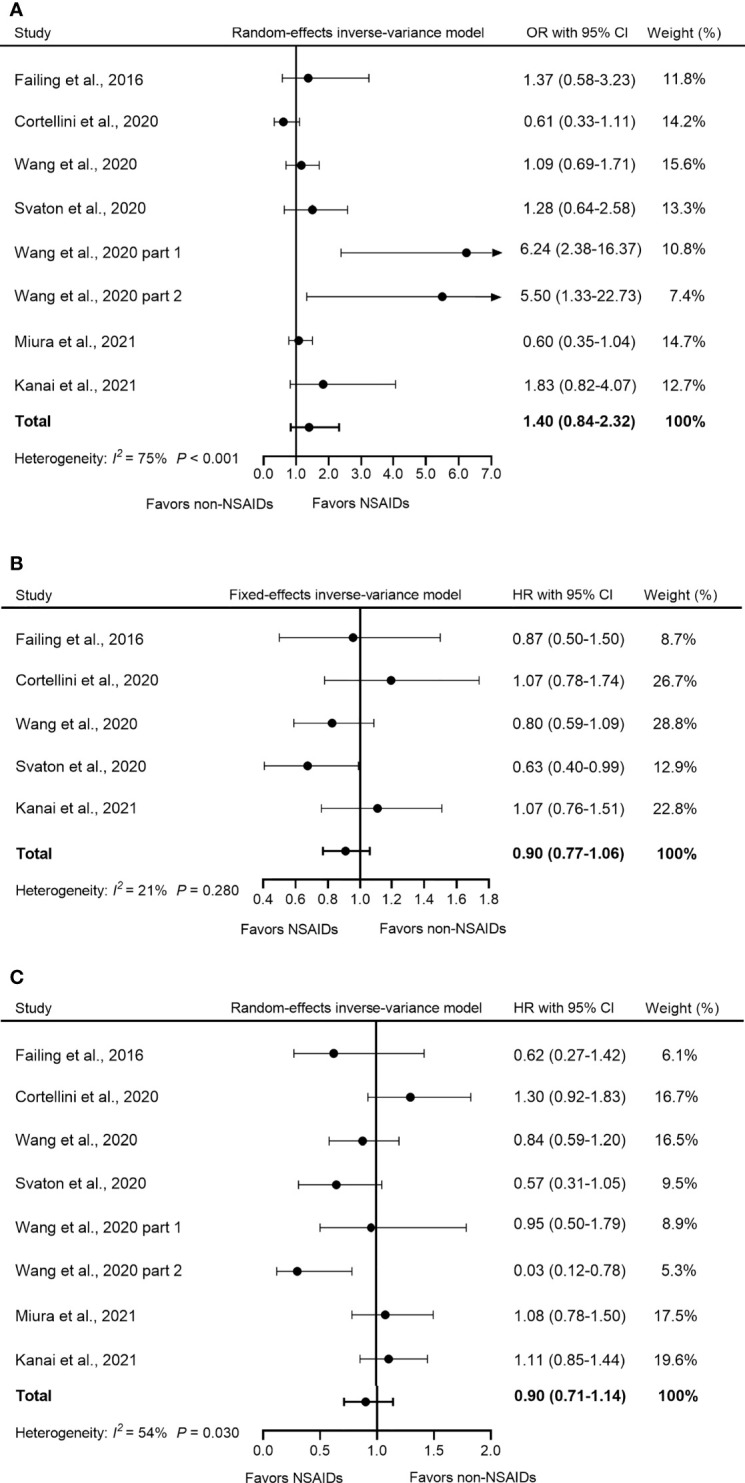
Forest plot of the correlation between concomitant use of NSAIDs and **(A)** ORR, **(B)** PFS, and **(C)** OS in patients receiving ICIs. The study of Wang et al. (2020) included two parts: melanoma and NSCLC with reported OR and OS respectively, so we named them “Wang et al., 2020 part 1” and “Wang et al., 2020 part 2”. The pooled OR of ORR was 1.40 (95% CI = 0.84–2.32, *p* = 0.190) and the random-effects model was adopted. The pooled HR of PFS was 0.90 (95% CI = 0.77–1.06, *p* = 0.186) and the fixed-effects model was adopted. The combined HR of OS was 0.90 (95% CI = 0.71–1.14, *p* = 0.384) and the random-effects model was adopted. By definition, OR > 1 or HR < 1 implied a better prognosis for NSAID users. NSAIDs, non-steroidal anti-inflammatory drugs; ORR, objective response rate; PFS, progression-free survival; OS, overall survival; OR, odds ratio; HR, hazard ratio; CI, confidence interval; ICIs, immune checkpoints inhibitors.

Seven studies evaluated the effect of concomitant use of NSAIDs on ORR in patients receiving ICI treatment. A highly significant heterogeneity was observed in these studies (*I* ^2^ *=* 75%, *Q*-test *p* < 0.001); thus, the random-effects model was adopted for analysis. The pooled OR showed that the use of NSAIDs did not significantly influence ORR in patients during ICI treatment (OR = 1.40, 95% CI = 0.84–2.32, *p* = 0.190) (**Figure 3A**).

Five studies reported PFS data. Our analysis showed that compared to non-NSAID users, NSAID use did not significantly influence PFS among NSAID users (HR = 0.90, 95% CI = 0.77–1.06, *p* = 0.186) (**Figure 3B**). Furthermore, no significant heterogeneity was detected in all five studies (*I* ^2^ *=* 21%, *Q*-test *p* = 0.210).

OS data were available in seven studies. Due to the high heterogeneity (*I* ^2^ *=* 54%, *p* = 0.030), a random-effects model was used for the analysis. The pooled HR data showed that the concomitant use of NSAIDs was not significantly associated with OS in patients treated with ICIs (HR = 0.90, 95% CI = 0.71–1.14, *p* = 0.384) (**Figure 3C**).

### Subgroup Analysis

A further subgroup analysis was performed according to the following variables: cancer type (NSCLC and melanoma) and ICI type (anti-CTLA-4, anti-PD-1, and anti-PD-L1). The results are presented in **Table 2**. These were basically consistent with the results of the entire population: opioid use during ICI treatment was significantly associated with a poor prognosis, while NSAID use did not influence prognosis in ICI-treated patients. In particular, NSAIDs were significantly associated with better PFS in patients in the anti-PD-1 subgroup (HR = 0.74, 95% CI = 0.58-0.96, *p* = 0.020).

**Table 2:** Results of subgroup analysis.

Concomitant medications	Analysis		ORR				PFS				OS		
			Association		Heterogeneity		Association		Heterogeneity		Association		Heterogeneity
		N	OR (95% CI)	*P*	*I^2^*	N	HR (95% CI)	*P*	*I^2^*	N	HR (95% CI)	*P*	*I^2^*
Opioids	**Total**	6	0.49 (0.37-0.65)	**<0.001**	32%	4	1.61 (1.37-1.89)	**<0.001**	0%	6	1.67 (1.30-2.14)	**<0.001**	69%
	**Cancer type**												
	NSCLC	2	0.48 (0.28-0.84)	**0.010**	59%	2	2.04 (1.37-3.03)	**<0.001**	0%	2	2.75 (1.94-3.91)	**<0.001**	0%
	Melanoma	–	–	–	–	–	–	–	–	–	–	–	–
	**ICIs type**												
	Anti-CTLA-4	–	–	–	–	–	–	–	–	–	–	–	–
	Anti-PD-1	2	0.48 (0.28- 0.84)	**0.010**	59%	1	1.93 (1.02-3.65)	**0.040**	0%	2	1.91 (1.21- 3.01)	**0.005**	68%
	Anti-PD-L1	–	–	–	–	–	–	–	–	–	–	–	–
NSAIDs	**Total**	8^*^	1.40 (0.84- 2.32)	0.190	75%	5	0.90 (0.77-1.06)	0.186	21%	8^*^	0.90 (0.71-1.14)	0.384	54%
	**Cancer type**												
	NSCLC	4	1.41 (0.65- 3.06)	0.380	73%	2	0.84 (0.50-1.41)	0.510	70%	4	0.80 (0.52-1.22)	0.310	72%
	Melanoma	3	1.97 (0.74-5.25)	0.170	81%	2	0.82 (0.62-1.07)	0.164	0%	3	0.83 (0.62-1.11)	0.211	0%
	**ICIs type**												
	Anti-CTLA-4	1	1.37 (0.58- 3.23)	0.470	0%	1	0.87 (0.50-1.51)	0.620	0%	1	0.62 (0.27-1.42)	0.260	0%
	Anti-PD-1	3	0.93 (0.60-1.44)	0.740	46%	2	0.74 (0.58-0.96)	**0.020**	0%	3	0.87 (0.63- 1.18)	0.360	43%
	Anti-PD-L1	–	–	–	–	–	–	–	–	–	–	–	–

ORR, objective response rate; PFS, progression-free survival; OS, overall survival; HR, hazard ratio; NSCLC, non-small cell lung cancer; NSAIDs, non-steroidal anti-inflammatory drugs; NOS, Newcastle–Ottawa Scale.Annotation: *The study by Wang SJ et al. included two parts and showed the HR and 95% CI respectively, and the total number refers to cohorts rather than studies.

### Sensitivity Analysis

Regarding the comparison between opioid users and nonopioid users, the pooled ORs for ORR were stable in the sensitivity analysis, ranging from 0.42 [95% CI = 0.30–0.50, after excluding the study by Cortellini et al., 2020 (35)] to 0.55 [95% CI = 0.39–0.78, after excluding the study by Gaucher et al., 2021 (29)] (**Figure S1A**). The pooled HRs for PFS were also stable in the sensitivity analysis, ranging from 1.56 [95% CI = 1.32–1.85, after excluding Iglesias−Santamaría et al., 2020 (28)] to 1.82 (95% CI = 1.44–2.30, after excluding Botticelli et al., 2021) (**Figure S1B**). In addition, the pooled HRs for OS did not change significantly in the sensitivity analysis and ranged from 1.52 [95% CI = 1.24–1.86, after excluding Iglesias−Santamaría et al., 2020 (28)] to 1.81 [95% CI = 1.36–2.41, after excluding Botticelli et al., 2021 (26)] (**Figure S1C**).

Comparison of NSAID users and non-NSAID users showed that the pooled ORs for ORR were stable in the sensitivity analysis, ranging from 1.11 (95% CI = 0.74–1.68, after excluding Wang et al., 2020 part 1) to 1.61 [95% CI = 0.93–2.87, after excluding Cortellini et al., 2020 (35) or 95% CI = 0.94–2.77, after excluding Miura et al., 2021 (31)] (**Figure S2A**). The pooled HR for PFS was also stable, ranging from 0.85 [95% CI = 0.70–1.03, after excluding Cortellini et al., 2020 (35)] to 0.95 (95% CI = 0.78–1.15, after excluding Wang et al., 2020 or 95% CI = 0.80–1.13, after excluding Svaton et al., 2020) (**Figure S2B**). Similarly, the pooled HRs for OS did not significantly influence the sensitivity analysis, ranging from 0.68 (95% CI = 0.90–1.19, after excluding Wang et al., 2020) to 0.99 (95% CI = 0.82–1.19, after excluding Wang et al., 2020 part 2) (**Figure S2C**).

### Publication Bias Analysis

Funnel plots were used to determine whether there was evidence of publication bias for pooled HRs for ORR, PFS, or OS analysis. In general, the funnel plots were distributed symmetrically, and the publication bias was modest (**Figures S3A–F**).

## Discussion

DDIs represent a key area of interest in the context of the urgent need to accelerate the selection process for distinguishing patients who will benefit from ICI therapy. Of all the medications that cancer patients require on a daily basis, analgesics represent a considerable proportion (14). However, the potential effect of ICI interactions with analgesics on alleviating cancer pain remains undetermined. In this study, we found that the use of opioids during ICI treatment showed an adverse effect on the prognosis of patients, while the concomitant use of NSAIDs could not significantly influence the prognosis in patients receiving ICIs.

Opioids are feasible analgesics for severe pain; they act by activating the μ opioid receptor (MOR), which results in a decrease in afferent nociceptive neuronal depolarization, thus producing the analgesic effect (41). However, many preclinical studies have reported that the interaction between opioids and MOR can affect the development of multiple cancers through different mechanisms. Morphine (an opioid)-induced phosphorylation of the epidermal growth factor receptor (EGFR) occurs *via* MOR in NSCLC cell lines, facilitating tumor proliferation and invasion (16). Morphine can also activate the MAPK/ERK signaling pathway in microvascular endothelial cells, which stimulates angiogenesis of breast tumors (17). Furthermore, in colon cancer, morphine can induce the secretion of urokinase plasminogen activator (uPA) that plays a crucial role in the degradation of the extracellular matrix, facilitating tumor invasion and metastasis. Opioid antagonists such as naloxone can reverse morphine-induced upregulation of uPA (42). In summary, opioids can directly promote tumor growth, which could impair the efficacy of ICIs.

In addition to the intrinsic traits of tumors, the efficacy of ICIs also depends on anticancer immunity. Opioids are potentially an incentive to the immunosuppressive tumor microenvironment (TME), which can be an impediment to treatment with ICIs. An *in vitro* study has shown that morphine can block IL-2 transcription, an iconic cytokine involved in the activation of CD8^+^ T cells (18). Furthermore, opioids such as morphine and β-endorphin can induce a significant increase in cAMP, which ultimately blocks the initiation of T-cell receptor signaling and results in impairment of CD8^+^ T cell function in the activation stage (18). In terms of antigen-presenting cell (APC) function in T cells, morphine can downregulate major histocompatibility complex class II expression, which inhibits the activation and proliferation of the CD4^+^ T cells. Inactivation of CD4^+^ T cells will further cause a decrease in the secretion of IL-2 and IFN-γ, impairing cytotoxic T lymphocyte-mediated tumor killing activity (43). Not all immune cells are conducive to the anticancer response, such as Tregs (44). Cornwell et al. demonstrated that long-term exposure to morphine can upregulate circulating Tregs (CD25^+^Foxp3^+^) levels in peripheral blood mononuclear cell samples by approximately five times in the rhesus monkey (19). In addition, another study also showed that the number of Tregs in breast cancer patients who have undergone surgery and were treated with sufentanil or fentanyl (belonging to opioids) increased significantly after 7 days (45).

There is convincing evidence supporting a link between gut dysbiosis and the efficacy of ICIs. Both quantitative and qualitative imbalances in the microbiota can potentially decrease the patient’s response to ICIs (46, 47). Long-term use of opioids has been definitely associated with gastrointestinal side effects, including constipation, bloating, nausea, and vomiting (41). Specifically, opioids can suppress protective mucus and bicarbonate secretion from the intestinal epithelium and weaken coordinated myenteric activity, thus delaying transit time and potentially increasing the risk of bacterial translocation in the human body (20). *In vivo* and *in vitro*, morphine has been shown to destroy the intestinal epithelial integrity by damaging the distribution of tight junction protein (ZO-1) in intestinal epithelial cells. As a result, the risk of *Escherichia coli* bacteria translocation to the mesenteric lymph nodes of mice increases after morphine treatment, inducing damage to the immune system (21). Furthermore, chronic morphine treatment can significantly alter the intestinal microbiota composition and induce a prominent proliferation of pathogenic Gram-positive bacteria and a decrease in bile-deconjugating bacterial strains. Intriguingly, morphine-induced microbial dysbiosis and intestinal barrier destruction can be rescued by transplanting the placebo-treated microbiota into morphine-treated animals (48).

Contrary to opioids, NSAIDs probably play a role in the inhibition of malignancies. The analgesic action of NSAIDs, particularly selective COX-2 inhibitors, has been explained on the basis of their inhibition of enzymes that synthesize prostaglandin E2 (PGE2) (49). Substantial evidence from preclinical studies has shown that overexpression of COX-2/PGE2 in multiple cancers is associated with many malignant phenotypes. In NSCLC, PGE2 can bind to the EP3 receptor, which promotes EGFR translocation. EGFR entering the nucleus can promote the expression of c-myc, cyclin D1, and PTGS2, and can contribute to tumor cell proliferation (22). Furthermore, COX-2/PGE2 can upregulate the expression of vascular endothelial growth factor receptor-1 in colon cancer by binding to EP3. This process can increase tumor angiogenesis and metastasis (23). In addition, COX-2/PGE2 can upregulate β1-integrin expression, to facilitate the invasion and migration of tumor cells (50). Consequently, NSAIDs are potentially able to favor cancer prophylaxis and regression by inhibiting COX-2/PGE2, which may partly explain why NSAIDs may improve survival in patients receiving ICIs to a certain extent compared to opioids.

In addition to directly regulating tumor progression, COX-2/PGE2 can also mediate reprogramming of the TME, leaving the TME in an immunosuppressive state. The malate–aspartate shuttle (MAS) system is critical to maintaining the redox equilibrium between mitochondria and cytoplasm in various cells (51). COX-2/PGE2 can seriously damage the MAS system in CD8^+^ T cells. As a result, there is a marked decrease in the content of aspartic acid and of various enzymes in the MAS system, resulting in the growth arrest of CD8^+^ T cells (24, 25). This might represent the key mechanism by which COX-2/PGE2 downregulates CD8^+^ T cells in the TME. COX-2/PGE2 can also inhibit the secretion of CCL5 and XCL1 by natural killer (NK) cells and the expression of CCR5 and XCR1 in conventional type 1 dendritic cells (cDC1), which can impair the function of NK cells and the accumulation of cDC1 in the TME, which are responsible for tumor immunity (24). Furthermore, downregulation of RIPK3 in myeloid-derived suppressor cells (MDSCs) can promote the activation of the COX-2/PGE2 axis and can generate a large amount of PGE2, which promotes the polarization of MDSCs to M2-type macrophages. At the same time, PGE2 can further reduce RIPK3 levels, forming a positive feedback loop to further promote the immunosuppressive activity of MDSCs (52). Taken together, NSAIDs, as inhibitors of COX-2/PGE2, are potentially able to reverse the immunosuppressive TME by increasing the infiltration of CD8^+^ T cells or other killer cells and by suppressing the function of MDSCs. Therefore, compared to opioids, NSAIDs and ICIs have synergistic effects, which may increase the therapeutic response of patients to ICIs.

Studies evaluating NSAIDs have also reported a lack of beneficial effect of concomitant use with ICIs [such as Miura et al., 2021 (31), Kanai et al., 2021 (30)], whereas Svaton et al. (2020) (33) reported higher ORR and longer OS and PFS in NSAID users, although the differences in most results were not statistically significant. A possible explanation is that the time of administration of NSAIDs may have affected the results. In Svaton et al. (33), patients started taking NSAIDs 1 month before treatment with ICIs. However, NSAIDs were used in Miura et al. (31) only at the start of ICI treatment. This suggests that only the prolonged administration of NSAIDs may improve the efficacy of treatment with ICIs. Clinicians should reasonably control the duration of NSAID treatment when combined with ICIs. Furthermore, NSAID users in Kanai et al. (30) had a higher prevalence of bone metastasis. It has been reported that bone metastasis can hinder the development of T cells and, thus, undermine the efficacy of ICI treatment (53). Therefore, future research on NSAIDs should give greater consideration to the effects of cancer pain associated with bone metastasis on outcome.

To our knowledge, this is the first meta-analysis to assess the influence of analgesics on the treatment of ICIs. Our study provides some useful information to oncologists in their clinical practice. Chronic use of opioids should be limited or replaced with NSAIDs as much as possible, to prevent the negative impact on concomitant treatment with ICIs and improve survival rates. For patients experiencing severe pain that is inadequately treated with NSAIDs, proper management of opioids is crucial to balance a pain-free period without influencing the outcome of ICI treatment. Opioids with weak or no immune modulation, such as buprenorphine, oxycodone, hydromorphone, and tramadol should be given before morphine, fentanyl, or codeine, which possess powerful immunosuppressive effects (54). In addition, it is necessary to apply some biological agents to regulate the gut microbiota and increase the efficacy of ICI treatment during the period when opioids are applied.

Certainly, our study has some intrinsic limitations. First, all included studies were retrospective designs, possibly lacking scientific control over variables, which could have led to deviations between results and actual clinical practice. Second, all studies included in this meta-analysis were published in English, which may have introduced a certain degree of bias. Third, some important characteristics, including age, sex, PD-L1 expression, line of ICI treatment, and pain grade, were not included in the subgroup analysis due to unavailability, and may have affected the universality of our findings.

## Conclusion

In summary, this study revealed that concomitant use of opioids is associated with a poor prognosis in patients treated with ICIs, while use of NSAIDs did not alter the efficacy of ICI treatment. Our findings provide important information for balancing management of cancer pain relief and efficacy of ICI treatment.

## Data Availability Statement

The original contributions presented in the study are included in the article/**Supplementary Material**. Further inquiries can be directed to the corresponding authors.

## Author Contributions

HG and ZL designed the study. ZM and XJ performed literature search and wrote the original manuscript. PJ and QW conceived the project and prepared the figures and tables. YZ and YL participated in the drafting and editing of the manuscript. XF and MJ provided statistical analysis. LJ committed to interpreting the results. HG revised the manuscript. ZM and XJ revised the draft and approved the final version to be submitted. All authors had full access to all data, critically revised the paper, approved the final analysis, and took responsibility for all aspects of the work.

## Conflict of Interest

The authors declare that the research was conducted in the absence of any commercial or financial relationships that could be construed as a potential conflict of interest.

## Publisher’s Note

All claims expressed in this article are solely those of the authors and do not necessarily represent those of their affiliated organizations, or those of the publisher, the editors and the reviewers. Any product that may be evaluated in this article, or claim that may be made by its manufacturer, is not guaranteed or endorsed by the publisher.

## Supplementary Material

Supplementary Figure 1 Sensitivity analysis for the correlation between concomitant use of opioids and **(A)** ORR, **(B)** PFS, **(C)** OS in patients receiving ICIs. Pooled ORs or HRs with 95% CI of the remaining studies were presented with accordingly removed studies. By definition, OR>1 or HR<1 implied a better prognosis for opioids users. ORR, objective response rate; PFS, progression-free survival; OS, overall survival; OR, odds ratio; HR, hazard ratio; CI, confidence interval; ICIs, immune checkpoints inhibitors.

Supplementary Figure 2 Sensitivity analysis for the correlation between concomitant use of NSAIDs and **(A)** ORR, **(B)** PFS, **(C)** OS in patients receiving ICIs. Pooled ORs or HRs with 95% CI of the remaining studies were presented with accordingly removed studies. By definition, OR>1 or HR<1 implied a better prognosis for NSAIDs users. NSAIDs, non-steroidal anti-inflammatory drugs; ORR, objective response rate; PFS, progression-free survival; OS, overall survival; OR, odds ratio; HR, hazard ratio; CI, confidence interval; ICIs, immune checkpoints inhibitors.

Supplementary Figure 3 Publication bias. Analysis for studies evaluating opioids: **(A)** Begg’s funnel plot of OR of ORR; Begg’s funnel plot of HR of **(B)** PFS and **(C)** OS. Analysis for studies evaluating NSAIDs: **(D)** Begg’s funnel plot of OR of ORR; Begg’s funnel plot of HR of **(E)** PFS and **(F)** OS. NSAIDs, non-steroidal anti-inflammatory drugs; ORR, objective response rate; PFS, progression-free survival; OS, overall survival; OR, odds ratio; HR, hazard ratio.
