# Supplementary figures and images for: Effect of Concomitant Use of Analgesics on Prognosis in Patients Treated With Immune Checkpoint Inhibitors: A Systematic Review and Meta-Analysis
Source: Front Immunol. 2022 May 6;13:861723. doi: 10.3389/fimmu.2022.861723 (PMC9120587; doi:10.3389/fimmu.2022.861723)

**A**

**B**

**Figure S1**

**C**

**A**

**B**

**Figure S2**

**C**


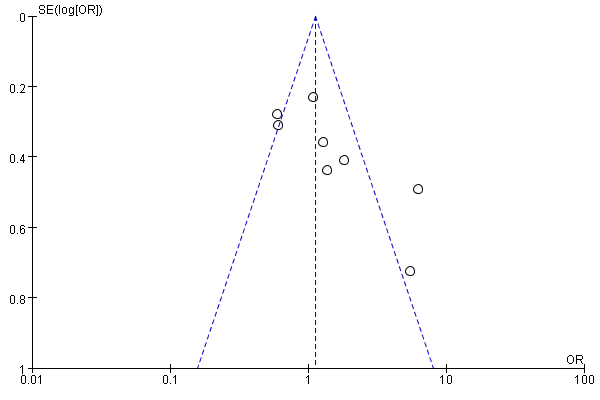

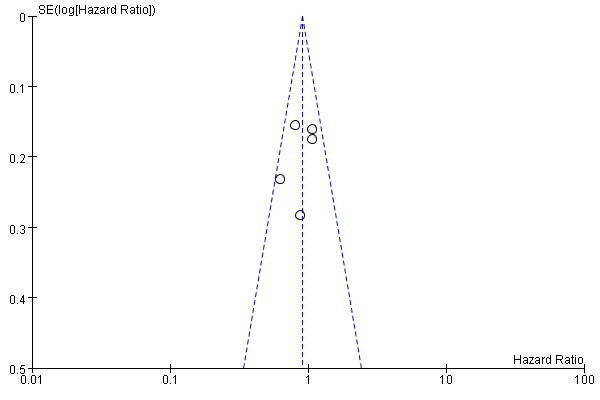

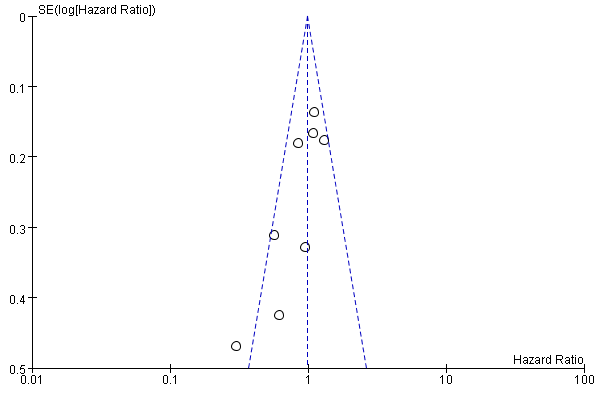

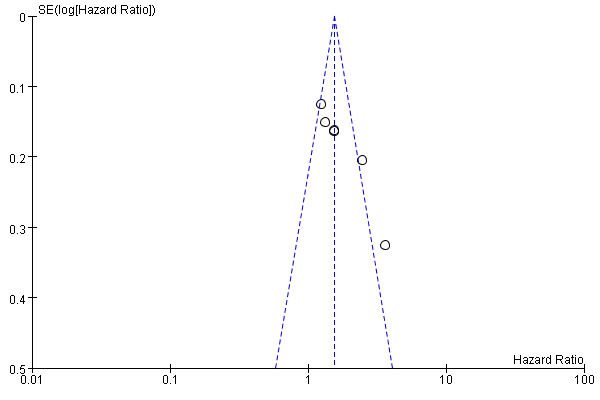

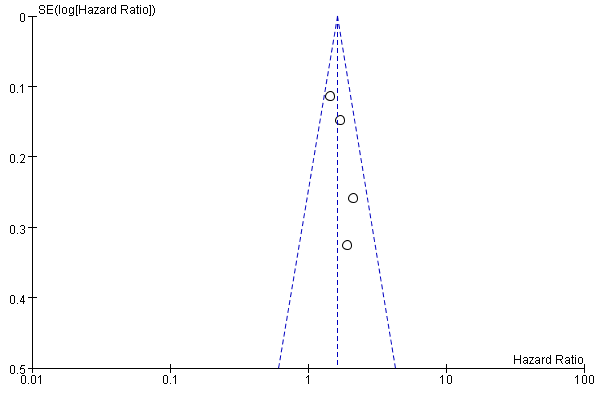

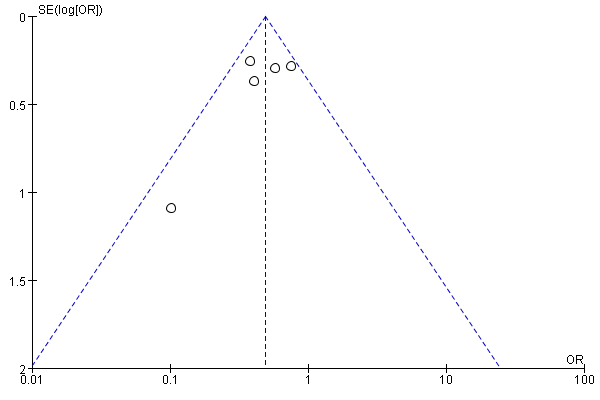


**Figure S3**

**F**

**E**

**D**

**C**

**B**

**A**

Supplement: Supplementary Figure 1 — Sensitivity analysis for the correlation between concomitant use of opioids and (A) ORR, (B) PFS, (C) OS in patients receiving ICIs. Pooled ORs or HRs with 95% CI of the remaining studies were presented with accordingly removed studies. By definition, OR>1 or HR<1 implied a better prognosis for opioids users. ORR, objective response rate; PFS, progression-free survival; OS, overall survival; OR, odds ratio; HR, hazard ratio; CI, confidence interval; ICIs, immune checkpoints inhibitors. [file DataSheet_1.docx]
