# Supplementary material for: Effect of Concomitant Use of Analgesics on Prognosis in Patients Treated With Immune Checkpoint Inhibitors: A Systematic Review and Meta-Analysis
Source: Front Immunol. 2022 May 6;13:861723. doi: 10.3389/fimmu.2022.861723 (PMC9120587; doi:10.3389/fimmu.2022.861723)
Supplement: Supplementary file 2 [file Table_1.docx]

**Table S1: Literature search criteria.**

| **PubMed Search: 126**  (((((((((((((neoplasms[Title/Abstract]) OR (neoplasm[Title/Abstract])) OR (cancer[Title/Abstract])) OR (tumor[Title/Abstract])) OR (malignancy[Title/Abstract])) OR (malignancies[Title/Abstract])) OR (carcinoma[Title/Abstract])) OR (melanoma[Title/Abstract])) OR (leukemia[Title/Abstract])) OR (leucocythaemia[Title/Abstract])) OR (leucocythemia[Title/Abstract])) OR (lymphoma[Title/Abstract])) AND (((((((((((((((((((((((((((((((((((((((((((((((((((((((pembrolizumab[Title/Abstract]) OR (lambrolizumab[Title/Abstract])) OR (Keytruda[Title/Abstract])) OR (MK-3475[Title/Abstract])) OR (nivolumab[Title/Abstract])) OR (MDX-1106[Title/Abstract])) OR (ONO-4538[Title/Abstract])) OR (BMS-936558[Title/Abstract])) OR (Opdivo[Title/Abstract])) OR (atezolizumab[Title/Abstract])) OR (MPDL3280A[Title/Abstract])) OR (Tecentriq[Title/Abstract])) OR (RG7446[Title/Abstract])) OR (RG 7446[Title/Abstract])) OR (camrelizumab[Title/Abstract])) OR (SHR-1210[Title/Abstract])) OR (SHR-1210[Title/Abstract])) OR (durvalumab[Title/Abstract])) OR (MEDI4736[Title/Abstract])) OR (MEDI-4736[Title/Abstract])) OR (Imfinzi[Title/Abstract])) OR (toripalimab[Title/Abstract])) OR (sintilimab[Title/Abstract])) OR (IBI 308[Title/Abstract])) OR (IBI308[Title/Abstract])) OR (IBI-308[Title/Abstract])) OR (tislelizumab[Title/Abstract])) OR (ipilimumab[Title/Abstract])) OR (Yervoy[Title/Abstract])) OR (MDX 010[Title/Abstract])) OR (MDX010[Title/Abstract])) OR (MDX-010[Title/Abstract])) OR (MDX-CTLA-4[Title/Abstract])) OR (MDX CTLA 4[Title/Abstract])) OR (immune checkpoint inhibitors[Title/Abstract])) OR (ICIs[Title/Abstract])) OR (immune checkpoint blockade[Title/Abstract])) OR (ICB[Title/Abstract])) OR (anti-PD1[Title/Abstract])) OR (PD-1[Title/Abstract])) OR (Programmed Death 1[Title/Abstract])) OR (Programmed Cell Death 1 Receptor[Title/Abstract])) OR (PD 1[Title/Abstract])) OR (PD1[Title/Abstract])) OR (Programmed Death-Ligand 1[Title/Abstract])) OR (PD-L1[Title/Abstract])) OR (programmed cell death 1 ligand 1 protein[Title/Abstract])) OR (PD L1[Title/Abstract])) OR (PDL1[Title/Abstract])) OR (CTLA-4[Title/Abstract])) OR (CD152[Title/Abstract])) OR (CTLA-4 Protein[Title/Abstract])) OR (CTLA 4 Protein[Title/Abstract])) OR (Cytotoxic T-Lymphocyte Antigen 4[Title/Abstract])) OR (Cytotoxic T Lymphocyte Antigen 4[Title/Abstract]))) AND (((((((((((((((((NSAID[Title/Abstract]) OR (NSAIDs[Title/Abstract])) OR (Nonsteroidal Anti-Inflammatory Agent[Title/Abstract])) OR (Nonsteroidal Anti Inflammatory Agent[Title/Abstract])) OR (Nonsteroidal Antiinflammatory Agents[Title/Abstract])) OR (Non-Steroidal Anti-Inflammatory Agents[Title/Abstract])) OR (Non Steroidal Anti Inflammatory Agents[Title/Abstract])) OR (Nonsteroidal Anti-Inflammatory Agents[Title/Abstract])) OR (Nonsteroidal Anti Inflammatory Agents[Title/Abstract])) OR (Non-Steroidal Anti-Inflammatory Agent[Title/Abstract])))) OR (cyclooxygenase inhibitors[Title/Abstract])) OR (cyclooxygenase 2 inhibitors[Title/Abstract])) OR (cyclooxygenase inhibitor[Title/Abstract])) OR (cox-2[Title/Abstract])) OR (((((((((((((((((((((((((Opioids[Title/Abstract]) OR (Opioid[Title/Abstract])) OR (Opioid Analgesics[Title/Abstract])) OR (Opioid Analgesic[Title/Abstract])) OR (Analgesic, Opioid[Title/Abstract])) OR (Partial Opioid Agonists[Title/Abstract])) OR (Agonists, Partial Opioid[Title/Abstract])) OR (Opioid Agonists, Partial[Title/Abstract])) OR (Opioid Partial Agonists[Title/Abstract])) OR (Agonists, Opioid Partial[Title/Abstract])) OR (Partial Agonists, Opioid[Title/Abstract])) OR (Full Opioid Agonists[Title/Abstract])) OR (Agonists, Full Opioid[Title/Abstract])) OR (Opioid Agonists, Full[Title/Abstract])) OR (Opioid Full Agonists[Title/Abstract])) OR (Agonists, Opioid Full[Title/Abstract])) OR (Full Agonists, Opioid[Title/Abstract])) OR (Opioid Mixed Agonist-Antagonists[Title/Abstract])) OR (Agonist-Antagonists, Opioid Mixed[Title/Abstract])) OR (Mixed Agonist-Antagonists, Opioid[Title/Abstract])) OR (Opioid Mixed Agonist Antagonists[Title/Abstract])) OR (Morphine[Title/Abstract])) OR (Codeine[Title/Abstract])) OR (Fentanyl[Title/Abstract])) OR (Tramadol[Title/Abstract]))) |
| --- |
| **Embase Search: 279**  ('neoplasms':ab,ti OR 'neoplasm':ab,ti OR 'cancer':ab,ti OR 'tumor':ab,ti OR 'malignancy':ab,ti OR 'malignancies':ab,ti OR 'carcinoma':ab,ti OR 'melanoma':ab,ti OR 'leukemia':ab,ti OR 'leucocythaemia':ab,ti OR 'leucocythemia':ab,ti OR 'lymphoma':ab,ti) AND ('pembrolizumab':ab,ti OR 'lambrolizumab':ab,ti OR 'keytruda':ab,ti OR 'mk-3475':ab,ti OR 'nivolumab':ab,ti OR 'mdx-1106':ab,ti OR 'ono-4538':ab,ti OR 'bms936558':ab,ti OR 'opdivo':ab,ti OR 'atezolizumab':ab,ti OR 'anti-pdl1':ab,ti OR 'mpdl3280a':ab,ti OR 'tecentriq':ab,ti OR 'rg7446':ab,ti OR 'camrelizumab':ab,ti OR 'shr-1210':ab,ti OR 'shr 1210':ab,ti OR 'durvalumab':ab,ti OR 'medi4736':ab,ti OR 'medi-4736':ab,ti OR 'imfinzi':ab,ti OR 'mdx 010':ab,ti OR 'sintilimab':ab,ti OR 'ibi 308':ab,ti OR 'ibi308':ab,ti OR 'ibi-308':ab,ti OR 'tislelizumab':ab,ti OR 'ipilimumab':ab,ti OR 'yervoy':ab,ti OR 'mdx010':ab,ti OR 'mdx-010':ab,ti OR 'mdx-ctla-4':ab,ti OR 'mdx ctla 4':ab,ti OR 'immune checkpoint inhibitors':ab,ti OR 'icis':ab,ti OR 'immune checkpoint blockade':ab,ti OR 'icb':ab,ti OR 'anti-pd1':ab,ti OR 'pd-1':ab,ti OR 'programmed death 1':ab,ti OR 'programmed cell death 1 receptor':ab,ti OR 'pd 1':ab,ti OR 'pd1':ab,ti OR 'programmed death-ligand 1':ab,ti OR 'pd-l1':ab,ti OR 'programmed cell death 1 ligand 1 protein':ab,ti OR 'pd l1':ab,ti OR 'pdl1':ab,ti OR 'ctla4':ab,ti OR 'cd152':ab,ti OR 'ctla-4 protein':ab,ti OR 'ctla 4 protein':ab,ti OR 'cytotoxic t-lymphocyte antigen 4':ab,ti OR 'cytotoxic t lymphocyte antigen 4':ab,ti) AND ('nsaid':ab,ti OR 'nsaids':ab,ti OR 'nonsteroidal anti-inflammatory agent':ab,ti OR 'nonsteroidal anti inflammatory agent':ab,ti OR 'nonsteroidal antiinflammatory agents':ab,ti OR 'non-steroidal anti-inflammatory agents':ab,ti OR 'non steroidal anti inflammatory agents':ab,ti OR 'nonsteroidal anti-inflammatory agents':ab,ti OR 'nonsteroidal anti inflammatory agents':ab,ti OR 'non-steroidal anti-inflammatory agent':ab,ti OR 'cyclooxygenase inhibitors':ab,ti OR 'cyclooxygenase 2 inhibitors':ab,ti OR 'cyclooxygenase inhibitor':ab,ti OR 'cox-2':ab,ti OR 'opioids':ab,ti OR 'opioid':ab,ti OR 'opioid analgesics':ab,ti OR 'opioid analgesic':ab,ti OR 'analgesic, opioid':ab,ti OR 'partial opioid agonists':ab,ti OR 'agonists, partial opioid':ab,ti OR 'opioid agonists, partial':ab,ti OR 'opioid partial agonists':ab,ti OR 'agonists, opioid partial':ab,ti OR 'partial agonists, opioid':ab,ti OR 'full opioid agonists':ab,ti OR 'agonists, full opioid':ab,ti OR 'opioid agonists, full':ab,ti OR 'opioid full agonists':ab,ti OR 'agonists, opioid full':ab,ti OR 'full agonists, opioid':ab,ti OR 'opioid mixed agonist-antagonists':ab,ti OR 'agonist-antagonists, opioid mixed':ab,ti OR 'mixed agonist-antagonists, opioid':ab,ti OR 'opioid mixed agonist antagonists':ab,ti OR 'morphine':ab,ti OR 'codeine':ab,ti OR 'fentanyl':ab,ti OR 'tramadol':ab,ti) |
| **Cochrane Library** **Search: 12**  (''neoplasms'' OR 'neoplasm'' OR ''cancer'' OR ''tumor'' OR ''malignancy'' OR ''malignancies'' OR ''carcinoma'' OR ''melanoma'' OR ''leukemia'' OR ''leucocythaemia'' OR ''leucocythemia'' OR ''lymphoma'') AND ("pembrolizumab" OR "lambrolizumab" OR "Keytruda" or "MK-3475" OR "nivolumab" OR "MDX-1106" or "ONO-4538" OR "BMS-936558" OR "Opdivo" OR "atezolizumab" OR "anti-PDL1" OR "MPDL3280A" OR "Tecentriq" OR "RG7446" or "RG-7446" or "camrelizumab" OR "SHR-1210" OR "SHR 1210" OR "durvalumab" OR "MEDI4736" OR "MEDI-4736" OR "Imfinzi" OR "MDX 010" OR "sintilimab" OR "IBI 308" OR "IBI308" OR "IBI-308" OR "tislelizumab" OR "ipilimumab" OR "Yervoy" OR "MDX010" OR "MDX-010" OR "MDX-CTLA-4" OR "MDX CTLA 4" OR "immune checkpoint inhibitors" OR "ICIs" OR "immune checkpoint blockade" OR "ICB" OR "anti-PD1" OR "PD-1" OR "Programmed Death 1" OR "Programmed Cell Death 1 Receptor" OR "PD 1" OR "PD1" OR "Programmed Death-Ligand 1" OR "PD-L1" OR "programmed cell death 1 ligand 1 protein" OR "PD L1" OR "PDL1" OR "CTLA-4" OR "CD152" OR "CTLA-4 Protein" OR "CTLA 4 Protein" OR "Cytotoxic T-Lymphocyte Antigen 4" OR "Cytotoxic T Lymphocyte Antigen 4") AND ("nsaid" OR "nsaids" OR "nonsteroidal anti-inflammatory agent" OR "nonsteroidal anti inflammatory agent" OR "nonsteroidal antiinflammatory agents" OR "non-steroidal anti-inflammatory agents" OR "non steroidal anti inflammatory agents" OR "nonsteroidal anti-inflammatory agents" OR "nonsteroidal anti inflammatory agents" OR "non-steroidal anti-inflammatory agent" OR "cyclooxygenase inhibitors" OR "cyclooxygenase 2 inhibitors" OR "cyclooxygenase inhibitor" OR "cox-2" OR "opioids" OR "opioid" OR "opioid analgesics" OR "opioid analgesic" OR "analgesic, opioid" OR "partial opioid agonists" OR "agonists, partial opioid" OR "opioid agonists, partial" OR "opioid partial agonists" OR "agonists, opioid partial" OR "partial agonists, opioid" OR "full opioid agonists" OR "agonists, full opioid" OR "opioid agonists" OR "opioid full agonists" OR "agonists, opioid full" OR "full agonists, opioid" OR "opioid mixed agonist-antagonists" OR "agonist-antagonists, opioid mixed" OR "mixed agonist-antagonists, opioid" OR "opioid mixed agonist antagonists" OR "morphine" OR "codeine" OR "fentanyl" OR "tramadol") |
