# Supplementary material for: Effect of Concomitant Use of Analgesics on Prognosis in Patients Treated With Immune Checkpoint Inhibitors: A Systematic Review and Meta-Analysis
Source: Front Immunol. 2022 May 6;13:861723. doi: 10.3389/fimmu.2022.861723 (PMC9120587; doi:10.3389/fimmu.2022.861723)
Supplement: Supplementary file 3 [file Table_2.docx]

**Table S2: The Newcastle-Ottawa scale quality assessment of included studies.**

| Study | Failing et al., 2016 | Iglesias‑Santamaría et al., 2020 | Cortellini et al., 2020 | Wang et al., 2020 | Svation et al., 2020 | Wang et al., 2020 | Taniguchi et al., 2020 | Botticelli et al., 2021 | Miura et al., 2021 | Gaucher et al., 2021 | Miura et al., 2021 |
| --- | --- | --- | --- | --- | --- | --- | --- | --- | --- | --- | --- |
| Representativeness of the exposed cohort | ★ | ★ | ★ | ★ | ★ | ★ | ★ | ★ | ★ | ★ | ★ |
| Selection of the non exposed cohort | ★ | ★ | ★ | ★ | ★ | ★ | ★ | ★ | ★ | ★ | ★ |
| Ascertainment of exposure | ★ | ★ | ★ | ★ | ★ | ★ | ★ | ★ | ★ | ★ | ★ |
| Demonstration that outcome of interest was not present at start of study | ★ | ★ | ★ | ★ | ★ | ★ | ★ | ★ | ★ | ★ | ★ |
| Comparability of cohorts on the basis of the design or analysis | ★ | ★★ | ★ | ★ | ★ | ★★ | ★ | ★ | ★★ | ★ | ★ |
| Assessment of outcome | ★ | ★ | ★ | ★ | ★ | ★ | ★ | ★ | ★ | ★ | ★ |
| Was follow-up long enough for outcomes to occur | - | ★ | ★ | ★ | ★ | ★ | ★ | ★ | - | - | ★ |
| Adequacy of follow up of corhort | - | ★ | ★ | - | ★ | ★ | - | ★ | - | - | - |
| Scores | 6 | 9 | 8 | 7 | 8 | 9 | 7 | 8 | 7 | 6 | 7 |
